# Expression of DNM3 is associated with good outcome in colorectal cancer

**DOI:** 10.1515/med-2022-0420

**Published:** 2022-01-24

**Authors:** Shao-ang Cheng, Xin Huang, Liang Jiang, Qi-Lian Liang, Xiao-Cui Hong, Hai-Xia Yang, Ke-Hui Hu, Xing-Bo Luo, Hui-Jie Zhang

**Affiliations:** Oncology Center, Affiliated Hospital of Guangdong Medical University, Zhanjiang, 524001, China; Interventional Ward, Affiliated Hospital of Guangdong Medical University, 57 People Avenue Zhanjiang, 524001, China

**Keywords:** dynamin3, colorectal cancer, clinicopathological characteristics

## Abstract

The aim of this study is to reveal the potential value of dynamin3 (DNM3) in colorectal cancer (CRC) evaluation of clinical diagnosis and prognosis. A total of 100 tissue samples were collected from 50 patients with stages I–IV, CRC tissues (*n* = 50) paired with non-cancerous adjacent colorectal tissues (*n* = 50). The expression levels of DNM3 were detected in 50 cases of CRC tissues and 50 cases of non-cancerous adjacent colorectal tissues by real-time fluorescent quantitative reverse transcription-polymerase chain reaction (RT-PCR). Immunohistochemical method (IHC) was conducted to semi-quantify the expression of DNM3 protein. Results showed that the relative expression of DNM3 mRNA in CRC tissues was 0.634-fold of that in non-cancerous adjacent colorectal tissues. The positive rate of DNM3 protein in CRC tissues (42.0%) was much lower than that in non-cancerous adjacent colorectal tissues (66.0%; *P* < 0.05). The expression level of DNM3 protein in CRC tissues was dependent on tumor size, degree of histological differentiation, and clinical stage (*P* < 0.05). The expression level of DNM3 mRNA in CRC tissues was significantly correlated with tumor size and pathology classification (*P* < 0.05). The research shows that detecting the expression of DNM3 helps in analyzing the tumor size, degree of histological differentiation, and clinical stage. Expression of DNM3 may be associated with good outcome in CRC.

## 1 Introduction

Globally, colorectal cancer (CRC) is the third most common malignancy and the second most common cause of death from malignancy. The WHO Cancer Research Center’s Globocan project estimated that there will be 1.8 million new cases of CRC and 880,000 deaths worldwide in 2018 [1]. The etiology of CRC is complex, and the exact molecular mechanism underlying its occurrence and development remains unclear. Tumor suppressor genes (TSGs) and their products have become the focus of research. An oncogene is a kind of gene that inhibits cell growth and has the potential to inhibit cancer. When inactivated, oncogenes may lead to malignant tumors. Studies showed that inactivation of multiple TSGs occurs during the development of CRC, and the inactivation mechanism includes loss of gene fragments, hypermethylation of gene promoters, and amplification of proto-oncogenes [2,3].

DNM3 is a TSG belonging to a highly conserved family of guanosine triphosphatase (GTP) molecules in biological evolution. It is involved in the formation of clathrin vesicles. Moreover, it is a signal transduction protein with GTP hydrolase activity. The molecule of DNM family proteins has five functional domains, namely, GTPase domain, intermediate domain, GTPase-effector domain, pleckstrin homology (PH) domain, and proline-rich domain (PRD) [4]. Among these domains, the PH domain can bind DNM to the cell membrane by binding to phosphatidylinositol lipids and interact with many different actin-associated proteins in the Src homologous-3 domain. Given the low affinity and weak specificity of the PH domain to negatively charged phosphatidylinositol lipids, many binding regions in the PRD domain can be connected to different functional regions of the cell membrane [5]. The structural features of DNM are widely involved in cellular functions, including foot process formation, plasma membrane and transmembrane vesicles, Golgi apparatus, network plate pseudopodia, phagocytosis, and cytokinesis [6,7,8,9]. Emerging evidence has shown that DNM3 is associated with tumor progression. Inokawa et al. have revealed that methylation of DNM3, which is downregulated by promoter methylation, predicts a poor prognosis for hepatocellular carcinoma (HCC) patients [10]. Numerous studies have reported that DNM3 inhibits the growth and metastasis of HCC by upregulating p53 expression or downregulating matrix metalloproteinase-2 MMP2 [10,11]. Similarly, DNM3 is found to play a tumor suppressive role in both colon and cervical cancers by regulating the activities of the MMP family [12,13]. However, our knowledge regarding the importance of DNM3 in tumor progression remains insufficient, and most knowledge has been obtained via observation. The precise molecular mechanism is not yet clearly understood.

In the present study, we analyzed the expression of DNM3 in CRC tissue samples and para-cancer tissue samples and its correlation with clinicopathological characteristics, and evaluated its potential clinical value in CRC based on previous studies, in the hope of providing new ideas for the diagnosis and/or treatment of CRC.

## 2 Materials and methods

### 2.1 Patients and tissue samples

Surgically removed specimens and para-tumorous colon tissues were gathered from 50 patients with CRC who underwent surgical treatment between January 2014 and January 2015 at the Affiliated Hospital of Guangdong Medical University, Guangdong, China. A total of 29 male and 21 female cases were included, with an age range of 32–81 years. The mean age was 58.4 ± 5.6 years. Among the cases, 37 were cases of CRC of the rectum and sigmoid colon, 11 cases were CRC of the right half colon, one case was CRC of descending colon and one case was CRC of the transverse colon. The eligibility criteria were as follows: (i) the patients had not suffered from a second primary cancer; (ii) the postoperative samples were confirmed by at least two pathologists; (iii) patients with CRC provided written informed consent; and (iv) the patients had not received any anticancer therapy prior to surgery. CRC tissues from the 50 patients and corresponding tissue adjacent to carcinoma specimens were collected through resection. 3–5 cm of the tissue adjacent to carcinoma was obtained from the intestine tissue. The pathology revealed no cancer lesions in colorectal tissue. Two samples were collected *in vitro* for 5 min. One sample was frozen with liquid nitrogen for 5 min and stored in a refrigerator at −80°C. The other specimen was fixed with 40 g/L buffer neutral formalin solution and embedded in conventional paraffin. The tumor size, number, presence of adhesion, lymph node metastasis, distant metastasis, and serum carcinoembryonic antigen (CEA) concentration were recorded.

### 2.2 Quantitative reverse transcription-polymerase chain reaction (RT-qPCR) assay

Total RNA was extracted from snap-frozen paired carcinoma and para-tumorous colon tissues by TRIzol reagent (Invitrogen, Thermo Fisher Scientific, MA, USA), and cDNA solution was synthesized using M-MLV reagent kit (Promega Bio, WI, USA) according to the manufacturer’s protocol. RT-qPCR was performed with SYBR^®^ Premix Ex Taq™ II (Takara Bio, Inc). The primers used in this study were as follows: β-actin forward, 5′-GGCGGCACCACCATGTACCCT-3′ and reverse, 5′-AGGGGCCGGACTCGTCATACT-3′; and DNM3 forward, 5′-AGTTCGCCTTGAGATTGAAGC-3′ and reverse, 5′-CGTGTGGGGAATAGACTCGTAAA-3′. The thermocycling conditions of qPCR were carried out at 95°C for 30 s, then a 2-step cycle procedure was used (at 95°C for 5 s and at 60°C for 30 s) for 40 cycles, with a final dissociation at 95°C for 15 s, 55°C for 30 s, and 95°C for 5 s. Data were quantified by the 2^−ΔΔCq^ method.

### 2.3 Immunohistochemical assay (IHC)

Tissues were formalin-fixed paraffin-embedded and 4 µm-thick sections were prepared. These sections were de-paraffinized twice in xylene for 10 min and then rehydrated with a gradient of ethanol solution. The antigens were exposed to citric acid buffer in microwave for 10 min, and endogenous peroxidase activity was blocked with PBS at room temperature for 10 min. The sections were incubated with the corresponding primary antibodies (anti-DNM3, 1:150, Abcam, USA) at 4°C overnight, followed by a secondary antibody (HRP AffiniPure Goat Anti-Rabbit IgG [H + L]. No. a21020, 1:1,000, AmyJet Scientific Inc, Wuhan, China) at room temperature for 30 min. Afterwards, color was developed with diaminobenzidine reagent (Boster Biological Technology, Ltd, Wuhan, China) for 10 min and hematoxylin for 2 min both at room temperature.

The immunoreactivity of DNM3 was evaluated as follows: five fields were randomly observed under a microscopy at 400× magnification (Olympus, Tokyo, Japan) and the percentage and intensities of immune-stained cells were calculated. The score was based on the relative staining area. <10%, 10–30%, 31–60%, and >61% of staining area were specified as 0, 1, 2, and 3, respectively. According to the strength of the immune-staining cells, another four-grade score was: 0, absent; 1, weak; 2, moderate; and 3, strong. The final staining score was calculated (area score × intensity score) as negative (0–2) or positive (≥3).

### 2.4 Statistical analysis

All statistical analyses were performed using SPSS version 21.0 software. The values were presented as the mean value ± standard error of the mean. Student’s *t*-test and chi-squared test were used to evaluate the significance of differences in laterality between the two groups. *P*-values of <0.05 were considered statistically significant.


**Ethical approval:** All procedures performed in studies involving human participants were in accordance with the ethical standards of the Ethics Committee at the Affiliated Hospital of Guangdong Medical University (PJ2014058KT) and with the 1964 Helsinki declaration and its later amendments or comparable ethical standards. The Ethics Committee at the Affiliated Hospital of Guangdong Medical University explicitly approved this study.

## 3 Results

**Figure 1:**
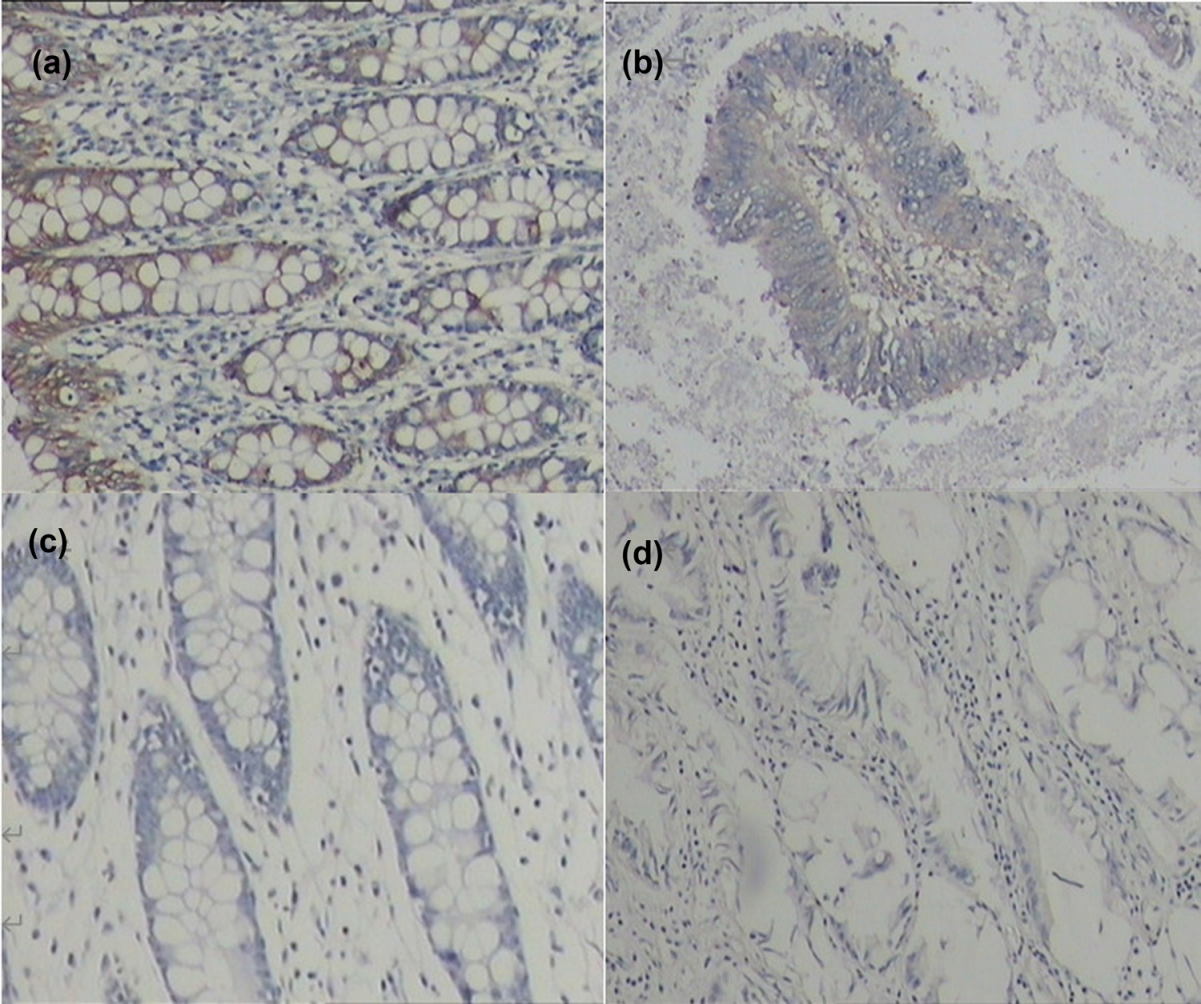
IHC staining of CRC and adjacent non-tumor tissues. (a) Positive expression of DNM3 in non-cancerous adjacent colorectal tissues; (b) positive expression of DNM3 in CRC tissues; (c) negative expression of DNM3 in non-cancerous adjacent colorectal tissues; (d) negative expression of DNM3 in CRC tissues. The positive area was stained with sappanwood purple.

The association between DNM3 expression and clinicopathological characteristics in CRC was determined. RT-PCR and IHC were used to detect the expression of DNM3 in CRC and non-cancerous adjacent colorectal tissues. The results showed that the relative expression of DNM3 mRNA in CRC was 0.634-fold of that in non-cancerous adjacent colorectal tissues (average mRNA expression: 0.634 ± 0.211 vs 1.000 ± 0.000; Table 1). The positive rate of DNM3 protein in CRC tissues (42.0%) was much lower than that in non-cancerous adjacent colorectal tissues (66.0%, *P* < 0.05; Figure 1 and Table 2).

**Table 1:** DNM3 expression in CRC and adjacent non-tumor colorectal tissues

Characteristic	DNM3 mRNA 2^−ΔΔCt^	*t*-value	*P*-value
CRC tissues	0.634 ± 0.211	14.974	<0.001
Adjacent non-tumor tissues	1.000 ± 0.000		

DNM3, dynamin3; CRC, colorectal cancer.

**Table 2:** Immunohistochemistry positive rates of DNM3

Rates	CRC tissues	Adjacent non-tumor tissues
Positive (*n*)	21	33
Negative (*n*)	29	17
Positive rate (%)	42.0	66.0
*χ* ^2^	5.722	
*P*-value	0.015	

DNM3, dynamin3; CRC, colorectal cancer.

The expression level of DNM3 mRNA in CRC tissues was independent of sex, age, clinical stage, portal vein tumor thrombus, and CEA concentration (*P* > 0.05) and significantly correlated with tumor size and pathology classification (*P* < 0.05; Table 3).

**Table 3:** Association between DNM3 expression and clinicopathological parameters in CRC

Variable	Cases	DNM3 mRNA 2^−ΔΔCt^	*t*-value	*P*-value
Age (years)				
<50	13	0.614 ± 0.148	0.489	0.627
≧50	37	0.621 ± 0.174		
Sex				
Male	29	0.613 ± 0.094	0.755	0.454
Female	21	0.594 ± 0.184		
Tumor size (cm)				
≦ 5	19	0.643 ± 0.117	2.342	0.024*
> 5	31	0.590 ± 0.139		
CEA (ng/mL)				
< 10	20	0.639 ± 0.088	0.075	0.941
≧ 10	30	0.615 ± 0.188		
TNM stage				
I–II	17	0.649 ± 0.153	0.573	0.570
III–IV	33	0.624 ± 0.172		
Differentiated degree				
Well/moderately	29	0.641 ± 0.055	11.451	<0.001*
Poorly	21	0.561 ± 0.091		

*P < 0.05.

The expression level of DNM3 protein in CRC tissues was dependent on tumor size, degree of histological differentiation, and clinical stage (*P* < 0.05) but not on sex, age, portal vein tumor thrombus, and CEA concentration (*P* > 0.05; Table 4).

**Table 4:** Association between DNM3 expression and clinicopathological parameters in CRC (detected by immunohistochemistry)

Variable	Cases	+	−	+%	*χ* ^2^	*P*-value
Age (years)						
<50	13	5	8	38.5	0.090	0.764
≧50	37	16	21	43.2		
Sex						
Male	29	14	15	48.3	1.116	0.291
Female	21	7	14	33.3		
Tumor size (cm)						
≤5	19	12	7	63.2	5.631	0.018*
>5	31	9	22	29.0		
CEA (ng/mL)						
<10	20	9	11	45.0	0.124	0.726
≧10	30	12	18	40.0		
TNM stage						
I–II	17	12	5	70.6	8.642	0.003*
III–IV	33	9	24	27.3		
Differentiated degree						
Well/moderately	29	16	13	55.2	4.918	0.027*
Poorly	21	5	16	23.8		

*P < 0.05.

## 4 Discussion

No previous studies have evaluated the role of clinical diagnosis of DNM3 on CRC. In this study, RT-qPCR and immunohistochemistry were performed, and DNM3 expression was found to be higher in adjacent non-tumor colorectal tissues than in CRC tissues. Numerous studies revealed that DNM3 can be served as an indicator to judge the severity and malignancy of the tumors [10,11,12,13]. For instance, in cervical invasive squamous cell carcinoma, Lee et al. found that inhibiting the expression of the DNM2 gene can promote the overexpression MMP2 (the main structural component of the basement membrane), leading to the easy passage of tumor cells through the basement membrane of epithelium, surrounding matrix, and into blood vessels or lymphatic vessels, as well as metastasis to other sites to form new tumor lesions [13]. Therefore, the expression of DNM2 can prevent tumor invasion and lymph node metastasis, and be used as a diagnosis indicator of 17 early cervical squamous cell carcinoma risk factors. Booken et al. found that TWIST1, a transcriptional regulatory factor that is highly expressed in peripheral blood nuclear cells of patients with Sezary syndrome, can upregulate DNM3 expression, indicating that DNM3 may play a role in the occurrence of T-cell lymphoma [14]. In addition, Shen et al. [15] first reported DNM3 expression in liver cancer tissue and found that DNM3 genes present in the tumor tissues of liver cancer is hypermethylated in the promoter region, whereas the adjacent normal tissues do not present methylation pattern. Inokawa et al. found a high level of methylated DNM3 gene promoter and low expression of DNM3 gene in 48 patients with liver cancer, as well as a negative correlation between DNM3 expression in liver cancer tissues and prognosis of the patients, suggesting that DNM3 behaves as a tumor suppressor gene [10]. The expression level in liver cancer tissues is negatively correlated with the prognosis of patients with liver cancer, possibly because methylated DNM3 can promote the expression of MMP2, facilitating the expansion of tumor cells through the basement membrane of the epithelium, invasion of surrounding stroma to enter blood vessels or lymphatic vessels, and metastasis to other sites to form new tumor lesions [10]. Zhang et al. found that DNM3 is poorly expressed in cancer tissues of patients with liver cancer with venous invasion and distant metastasis, whereas upregulated DNM3 expression can inhibit the proliferation and metastasis formation of liver cancer cells [11]. Jiang et al. found that DNM3 can regulate the expression of MMP-2 and MMP-9, weaken the malignant behavior of colon cancer, and promote colon cancer invasion and migration [12]. Review articles suggested that DNM3 might be a novel candidate gene for TSGs [16]. Therefore, DNM3 is expected to be a new target for the treatment of liver cancer. The possible mechanism is to arrest the cell cycle of liver cancer cells at the G0/G1 phase by upregulating the expression of p53 protein and promote the apoptosis of liver cancer cells to achieve the anti-tumor effect.

The present study detected DNM3 in CRC tissue samples and tissues adjacent to carcinoma specimens and examined its expression and clinicopathological characteristics. The results have shown that the positive rate of DNM3 protein expression was significantly correlated with tumor size, histological differentiation degree, and TNM stage (*P* < 0.05) but not with gender, age, and CEA concentration (*P* > 0.05). High tumor volume indicated a low positive rate of DNM3 protein expression. The positive rate of DNM3 protein expression was lower in patients with poorly differentiated degree than those with well or moderately differentiated degree, suggesting that level of DNM3 was reduced by the differentiation process. The positive rate for DNM3 protein expression in TNM staging III and IV was lower than that of staging I and II. The mRNA expression levels of DNM3 were negatively associated with tumor size and degree of histological differentiation (*P* < 0.05). No significant association was detected between DNM3 expression and other clinicopathological parameters, including sex, age, CEA, and TNM stage (*P* > 0.05). Therefore, DNM3 expression at both mRNA and protein levels in CRC tissues was lower than that of non-cancerous adjacent colorectal tissues.

In summary, our data revealed that DNM3 could be a promising clinical marker for CRC patients, monitoring the expression of DNM3 may be helpful in predicting the tumor size, TNM stage, and histological differentiation degree of CRC. Expression of DNM3 may be associated with good outcome in CRC.
